# Evolutionary Trajectories of *Avian Avulaviruses* and Vaccines Compatibilities in Poultry

**DOI:** 10.3390/vaccines10111862

**Published:** 2022-11-03

**Authors:** Mohammed A. Rohaim, Mohammad Q. Al-Natour, Rania F. El Naggar, Mohammed A. Abdelsabour, Yahia M. Madbouly, Kawkab A. Ahmed, Muhammad Munir

**Affiliations:** 1 Division of Biomedical and Life Sciences, Faculty of Health and Medicine, Lancaster University, Lancaster LA1 4YG, UK; 2 Department of Virology, Faculty of Veterinary Medicine, Cairo University, Giza 12211, Egypt; 3 Department of Veterinary Pathology & Public Health, Faculty of Veterinary Medicine, Jordan University of Science and Technology (JUST), P.O. Box 3030, Irbid 22110, Jordan; 4 Department of Virology, Faculty of Veterinary Medicine, University of Sadat City, Sadat 32897, Egypt; 5 Department of Poultry Viral Vaccines, Veterinary Serum and Vaccine Research Institute (VSVRI), Agriculture Research Centre (ARC), Cairo 11435, Egypt; 6 Department of Pathology, Faculty of Veterinary Medicine, Cairo University, Giza 12211, Egypt

**Keywords:** avian orthoavulaviruses 1, evolutionary pressure, vaccine, efficacy, Jordan

## Abstract

Newcastle disease virus (NDV) causes one of the highly infectious avian diseases in poultry leading to genuine financial misfortunes around the world. Recently, there has been an increasing trend in the number of ND-associated outbreaks in commercial Jordanian poultry flocks indicating a possible complex evolutionary dynamic of NDV infections in the country. To underpin the dynamics of circulating NDV strains and to assess the vaccine-escape potential, a total of 130 samples were collected from different poultry flocks in six Jordanian Governorates during 2019–2021. Twenty positive isolates, based on real-time reverse transcriptase PCR, were used for further genetic characterization and evolutionary analysis. Our results showed that there is a high evolutionary distance between the newly identified NDV strains (genotype VII.1.1) in this study and the commercially used vaccines (genotypes I and II), suggesting that circulating NDV field strains are under constant evolutionary pressure. These mutations may significantly affect flocks that have received vaccinations as well as flocks with insufficient immunity in terms of viral immunity and disease dynamics. To assess this further, we investigated the efficacy of the heterologous inactivated LaSota or homologous genotype VII.1.1 vaccine for their protection against virulent NDV in chicken. Vaccine-induced immunity was evaluated based on the serology, and protection efficacy was assessed based on clinical signs, survival rates, histopathology, and viral shedding. Chickens vaccinated with the inactivated genotype VII.1.1 based vaccine showed 100% protection with a significant reduction in virus shedding, and ameliorated histopathology lesions compared to LaSota vaccinated chicks that showed 60% protection. These results revealed that the usage of NDV inactivated vaccine from the circulating field strains can successfully ameliorate the clinical outcome and virus pathobiology in vaccinated chicks and will serve as an effective vaccine against the threat posed by commonly circulating NDV strains in the poultry industry.

## 1. Introduction

The Paramyxoviridae family consists of a large number of viruses which are isolated from a wide range of human and other animal species including measles, mumps, and respiratory syncytial viruses, Newcastle disease virus (NDV), canine distemper, and rinderpest viruses [1]. All paramyxoviruses are pleomorphic, enveloped, single stranded and non-segmented viruses containing a negative sense RNA genome of 10–17 Kb size. Based on structure, genomic organization, and sequence relatedness, this family is divided into two subfamilies: Paramyxovirinae and Pneumovirinae [2]. The Paramyxovirinae subfamily has five genera: *Respirovirus*, *Rubulavirus*, *Morbillivirus*, *Henipavirus*, and *Avulavirus*, whereas the Pneumovirinae subfamily has two genera: *Pneumovirus* and *Metapneumovirus* [3].

All paramyxoviruses isolated from avian species have been classified into two genera: *Avulavirus*, which represents avian paramyxoviruses (APMV), and *Metapneumovirus*, which represents avian pneumoviruses. Based on hemagglutination inhibition (HI) and neuraminidase inhibition (NI) assays, it has been concluded that avian avulaviruses infect a wide range of domestic and wild birds all over the world [4]. Avulaviruses’ RNA genomes encode six structural proteins (NP, P, M, F, HN, and L) as well as two non-structural proteins (V and W) via RNA editing [2,4]. The hemagglutinin-neuraminidase (HN) and fusion (F) proteins are surface glycoproteins found in avulaviruses. The new classification criterion has been proposed that involve the use of genomic sequence comparisons in the categorization of avulaviruses due to issues associated with cross-reactivity among some serotypes of avulaviruses in serologic tests. According to recent criterion, *Orthoavulavirus*, *Paraavulavirus*, and *Metaavulavirus* are three genera that now make up the Avulavirinae subfamily [1,5].

*Avian Avulavirus* serotype-1 (AAvV-1) is a member of the *Avulavirus* genus in the Paramyxoviridae family that causes ND in chicken. The antigenic serotypes that evolve in this group as a result of environmental or vaccination pressure elude the immune system of birds and are responsible for vaccine failure [6,7]. The fusion (F) and hemagglutinin neuraminidase (HN) genes, which encode for structural envelope proteins that play host recognition, infection, and pathogenesis roles, but also influence the antigenicity and immunogenicity of ND viruses have a high genetic and antigenic diversity within the AAvV-1 serotype [8,9,10]. Animal models (1-day-old *Gallus* chick) are used in infectivity assays for ND viruses, which are expensive and time consuming. Monobasic amino acid sequences at positions 112–113 and 115–116 of the C-terminus of the fusion protein cleavage site (FPCS) with leucine (L) at position 117 and/or intracerebral pathogenicity indices (ICPI) of 0.7 are used to designate low-virulent strains [11,12].

*Avian Metaavulavirus* has been known to cause disease, specifically mild respiratory infections in domestic poultry, including turkeys and chickens, and pose many economic effects on egg production and poultry industries [13]. The virus was first isolated from a strain in Yucaipa, California in 1956. Since then, other isolates of the virus have been isolated worldwide. Avian paramyxovirus 2 (APMV-2) has been isolated from a wide range of birds, including chickens, turkeys, racing pigeons and feral birds and appears to be circulating worldwide [1,14,15,16,17].

Furthermore, the prevalence of APMV-2 antibodies in several bird species, including commercial poultry, has been investigated [13,18,19]. Chickens, broilers, and layers from the United States, Canada, Russia, Japan, Israel, India, Saudi Arabia, the United Kingdom, and Costa Rica, as well as turkeys from the United States, Canada, Israel, France, and Italy, have all been shown to carry APMV-2 viruses. Infections with APMV-2 reduced turkey hatchability and poult output [20]. More significant illness, particularly in turkeys during subsequent infections, has been documented [21]. APMV-2 infections in turkey flocks have also been reported by virus isolation and the presence of antibodies [22]. The reason of the reduction in egg production was assumed to be APMV-2, which was identified from commercial layer farms and broiler breeder farms in Scotland [23]. APMV-2 infection in chickens via intramuscular and intratracheal routes produced no evident respiratory disease [24]. Similar findings were found in turkeys infected by intratracheal route [25].

Despite the widespread use of a LaSota and Hitchner B1-based vaccine in the poultry, ND outbreaks are still common in the Middle East, where the most commonly circulating NDV isolates are taxonomically categorized as genotype VII [26,27,28]. Because of their significant contribution to the ongoing ND pandemic, these genotype VII isolates are currently regarded as the most economically relevant NDV strains in Jordan and also frequently isolated among flocks that have been vaccinated with traditional genotype II-based ND vaccines. Because genotype mismatch between genotype II-based ND vaccines and the circulating genotype VII NDV are widely thought to be responsible for the current vaccines’ suboptimal protective efficacy. Therefore, development of new vaccines based on the currently prevalent genotype VII NDV has the potential to improve the effectiveness of ND control in the global poultry industry. 

The aim of this study is to detect and characterize AAvVs using genetic and antigenic techniques to provide insights into the ecology of these viruses. We demonstrate the presence of two different avian avulavirus serotypes: one has been previously described in chicken (AOAvV-1) and one distantly related to AAvV-2/APMV-2. Further, we demonstrated the vaccine efficacy of currently deployed heterologous vaccine and a newly developed homologous vaccine in chicken. The finding warrants continued surveillance of AOAvV-1 strains in poultry and to revise vaccines and vaccination strategies trained by the ground realities. 

## 2. Materials and Methods

### 2.1. Ethics Statement

Samples were collected by trained veterinarians. Samples processing and virus isolation procedures were carried out in strict accordance with the guidance and regulations of animal welfare and health that approved by the Department of Veterinary Pathology and Public Health, Faculty of Veterinary Medicine, Jordan University of Science and Technology (JUST), Jordan (JUST 387-2020).

### 2.2. Sampling History, Virus Isolation, and Biological Characterization

A total of 130 swab samples were collected from various Jordanian poultry flocks (Table 1). All samples were collected from vaccinated flocks except Turkey, Peacock, and Ostrich (Table 2). Individual swabs were collected in viral transport medium supplemented with antibiotics (isotonic PBS, 2000 U/mL penicillin, 2 mg/mL streptomycin, 50 μg/mL gentamycin, 50 U/mL nystatin, and 0.5% BSA). Swab samples were cleared by centrifugation for 5 min at 1700 rpm at 10 °C, and the supernatants were collected and stored at −80 °C until further use.

According to the OIE Manual of Standards for Diagnostic Tests and Vaccines, each sample was inoculated (in triplicate) into the allantoic sac of 9–10-day embryonated chicken eggs (ECEs) for viral isolation [29]. The positive HA samples were biologically characterized using intracerebral pathogenicity test in Rhode Island Red SPF chicks using the standard protocols [29]. All samples were negative for other avian respiratory viruses including influenza viruses and infectious bronchitis virus (IBV).

### 2.3. RNA Extraction, Genome Amplification and Sequencing

Samples were subjected to RNA extraction from the allantoic fluid using an RNA extraction kit (RNAeasy Mini Kit, Qiagen, Hilden, Germany) according to the manufacturer’s instructions. Detection of avian avulaviruses was conducted using a real-time reverse transcriptase PCR based on the M gene of NDV and the N gene of aMPV as described previously [30]. For full length fusion (F) gene amplification, RNA was reverse transcribed into cDNA using a Superscript IV First-Strand cDNA Synthesis Kit (Invitrogen, Waltham, MA, USA) and the second strand was synthesized with Q5 DNA Polymerase (New England Biolabs, Ipswich, MA, USA) using for amplification and sequencing of the full length F gene [31,32]. Amplified PCR products were visualized by electrophoresis on a 1.2% agarose gel electrophoresis and then purified using a QIAquick Gel Extraction Kit (Qiagen, Hilden, Germany) following the manufacturer’s instructions. The purified PCR products were sequenced bi-directionally with both sense and antisense primers that were used in the PCR amplification [31,32] using ABI PRISM BigDye Terminator version 3.1 (Applied Biosystems, Foster City, CA, USA) by Sanger sequencing method on a 3500 Applied Biosystems capillary sequencer (Source Bioscience, Cambridge, UK).

### 2.4. Sequence Analyses and Phylogeny

The F nucleotide and amino acid sequences were retrieved in fasta format from the NCBI GenBank and compared to those found in ViralZone UniProtKB/Swiss-Prot entries using corresponding accession numbers. These sequences were edited to the same length and aligned using ClustalW, which is included in BioEdit version 7.0. [33]. The obtained nucleotide sequences were submitted to GenBank and assigned accession numbers are outlined (Table 1). Sequence Demarcation Tool (SDT) was used to display the amino acid pairwise identity scores through a color-coded matrix [34]. 

Phylogenetic analyses were conducted using general time-reversible (GTR) model [35], which was selected using jModelTest [36], and maximum-likelihood trees were constructed using RaxML version 8.2.11 [37] with 1000 bootstrap replicates. The phylogenetic analysis was performed on nucleotides based on the pilot tree proposed by Dimitrov et al. [38] to maintain the tree topology and to ascertain the genotypes of avian avulaviruses isolates.

### 2.5. Mutations Mapping at Variable Positions and Functional Regions

The F nucleotide sequences were later translated into amino acid sequences in MEGA X program v10.1.8 to compare our AOAvV-1 isolates, and vaccine (LaSota: JF950510.1) strains at the amino acid levels. Sequence logos, graphical representations of patterns within the F protein aligned sequences, were generated using the WebLogo service (http://weblogo.threeplusone.com/create.cgi, accessed on 15 August 2022). Sequence logos give a fuller and more accurate representation of F protein sequence similarity than consensus sequences, and they can quickly expose important characteristics of the alignment that might otherwise be difficult to notice.

Sequences variations were mapped onto the protein structures and entropy calculations with the aid of Scop3D tool, which visualizes variations across multiple sequences on the protein structures [39]. The F protein numbering was based on LaSota using GenBank accession number JF950510.1. The functional regions were defined based on literature and were mapped on the structures and Jalview or Chimera-analyzed models for diversity as visualized to the predicted structure models.

### 2.6. DiscoTope: Structure Based Antibody Prediction

The interaction of antibodies with antigens is one of the most significant immune system strategies for removing pathogenic organisms from the host. Antibodies bind to antigens at B-cell epitopes. The precise placement of B-cell epitopes is critical in many scientific applications, including rational vaccine design, disease diagnostics, and immunotherapeutics. However, because experimental mapping of epitopes is time consuming, in silico approaches provide an interesting supplementary option [40]. Using the Discotope 2.0 online tool (https://services.healthtech.dtu.dk/service.php?DiscoTope-2.0, accessed on 11 August 2022), we tried to map the antibody binding sites within the F protein of NDV that will help to identify these residues compared to those in commercially used vaccines among Jordanian poultry sectors.

### 2.7. F Protein Structural Homology Analyses and Selective Pressure

To identify the conserved regions, homology models for the translated F proteins were created by matching sequences using multiple sequence alignment (MSA) with the help of ClustalW, which is included in the BioEdit software version 7.0 [33]. The consensus areas for each protein in the field isolates and vaccine were utilized in a BLAST search against the Protein Data Bank (PDB) to find known homologs or orthologs. The Synonymous-Non-Synonymous Analysis Program (SNAP) was used to predict the F gene-specific estimates of dN/dS [41]. The number of potential synonymous and non-synonymous changes as well as the number of actual synonymous and non-synonymous changes in codon between each pair were counted. Then, the dN/dS ratio was calculated by comparing the proportion of observed non-synonymous substitutions over the proportion of observed synonymous substitutions. These were then adjusted for multiple hits using the Jukes–Cantor correction [41]. A higher score than 0 indicates a more dominant diversifying positive selection while below 0 indicates negative selection.

### 2.8. Preparation of Inactivated Newcastle Disease Virus Vaccine

The genotype VII.1.1 seed virus (ON858797 AOAvV-1 isolate NDV/chicken/Jordan/MQA-N-13/2021) was propagated via inoculation into the allantoic sac of specific pathogen-free embryonated chicken eggs (SPF-ECE) which were incubated at 37 °C. Allantoic fluids from these inoculated eggs were harvested after overnight chilling at 4 °C and tested for hemagglutination using 1% chicken red blood cells (RBCs). The Egg Infective Dose 50 (EID_50_) was determined via titration in 10-day-old SPF-ECE according to the method described [42].

The titrated virus (ON858797 AOAvV-1 isolate NDV/chicken/Jordan/MQA-N-13/2021) was used as a master seed for the preparation of inactivated vaccine. Seed virus inactivation was conducted with formalin (final concentration of 0.1%) for 18 h at 37 °C. Complete inactivation of the virus was confirmed through three passages in 10-day-old embryonated SPF chicken eggs followed by HA and EID_50._ All SPF chicken embryos inoculated with formalin-treated virus remained alive after 120 hours, and no HA-based positivity was detected. The final dose used was 10^8^ EID_50_/0.5 mL per chick. Inactivated NDV vaccine was prepared as water in oil emulsion (W/O) using Montanide ISA 70 at a ratio of 3/7 (*v*/*v*) aqueous/oil ratio. The manufacturing process was carried out according to the standard protocol of SEPPIC, France. The prepared vaccine were tested for its sterility and safety according to OIE [29]. Stability testing of emulsion involves determination of stability at long-term storage at 4 °C and 25 °C [43,44]. Velogenic Newcastle disease virus (vNDV) ON858797 AOAvV-1 isolate NDV/chicken/Jordan/MQA-N-13/2021 strain was used to challenge the vaccinated and non-vaccinated (positive control) chicks.

### 2.9. Vaccination and Challenge Experiments

**Figure 1:**
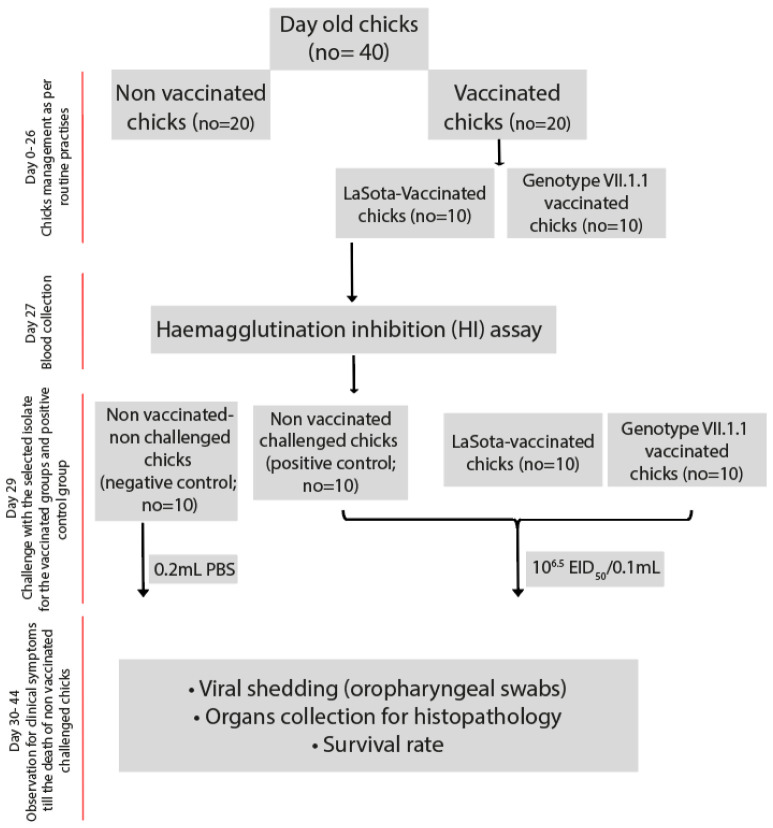
Experimental layout for the assessment of vaccines effectiveness in chicks.

Forty SPF Rhode Island Red chicks were housed separately in two groups: vaccinated (n = 20) and unvaccinated (n = 20). The challenge experiments were conducted in accordance with all relevant guidelines and animal ethics permits issued by Department of Veterinary Pathology & Public Health, Faculty of Veterinary Medicine, Jordan University of Science and Technology (JUST), Irbid, Jordan. Chicks in the unvaccinated group were divided into three subgroups: non-vaccinated challenged (positive control, n = 10) and non-vaccinated non-challenged (negative control, n = 10). However, chicks in the vaccinated group (n = 20) were kept in two groups (10 each); LaSota vaccinated challenged group and genotype VII.1.1 vaccinated challenged group (Figure 1) and administered either the inactivated LaSota vaccine (genotype II) or inactivated genotype VII.1.1 vaccine on day 7 at a dose of 0.5 mL per chick via the subcutaneous route around the neck region (Figure 1).

Chicks in the vaccinated- challenged and non-vaccinated challenged groups was challenged with a dose of 100 μL of 10^6.5^EID_50_ (ON858797 AOAvV-1 isolate NDV/chicken/Jordan/MQA-N-13/2021) strain through the oculonasal route on day 29. The mock-infected group served as a negative control and was inoculated with 100 μL of sterile PBS (Figure 1). For the next 10 days, chicks were monitored twice daily for any clinical signs including depression, sneezing/coughing, facial swelling, respiratory sounds, ocular/nasal discharge, ruffled feathers, dyspnea, greenish diarrhea, paralysis and tremors as well as necropsy lesions in dead chicks for pathognomic lesions of NDV including hemorrhages in the proventriculus and cecal tonsils.

### 2.10. Serology, Virus Shedding and Histopathology

Serum samples were obtained pre- (day 27) and post-challenge (day 38) from the vaccinated challenged groups and tested using an HI assay. The HI assay was performed using inactivated NDV antigen with 4 HAU in 0.025 mL [29]. Titers were calculated as the reciprocal of the highest serum dilution providing complete hemagglutination inhibition. Serum titers of 1:8 (2^3^) or lower were considered negative for antibodies against NDV. Virus shedding was detected using previously described assays for identification of velogenic strains of NDV in oropharyngeal swabs [29]. Oropharyngeal swabs were collected, placed in virus transport medium, filtered through a 0.2 µm filter and then aliquoted and stored at −70 °C until all samples were collected before analysis as previously described [29].

Selected tissues including trachea and lungs were collected, fixed by immersion in 10% neutral buffered formalin at room temperature for 48 h and followed by processing and embedding in paraffin wax. Tissue sections of 5 μm were stained with Hematoxylin and Eosin and examined for microscopic lesions under a light microscope.

### 2.11. Statistical Analysis

Pairwise comparisons of challenged (clinical and sub-lethal doses) and control groups (positive and negative) were performed using Student’s *t*-test. Kaplan–Meier analysis was performed to calculate the survival rates. Two-tailed Student’s *t*-test and one-way analysis of variance (ANOVA) were used to determine differences between groups. Statistical significance is shown with values of *p* < 0.05. All data were represented as the mean ± standard deviation (SD). Statistical analyses were conducted using GraphPad Prism 7 (GraphPad Software, La Jolla, CA, USA).

## 3. Results

### 3.1. NDV Epidemiology in Jordan and Biological Characterization

We present the isolation, biological characterization and genetic analyses that map the evolution of NDV in Jordan during 2019–2021. A total of 130 samples were individually screened by RT-qPCR, followed by full length amplifications for F gene of positive samples. From this screening, 20 out of 130 samples (20/130; 15.4%) were positive among all tested swab samples for AOAvV-1 (Table 1) while only two samples were positive for avian paramyxovirus 2 (2/130; 1.5%). The ICPI was conducted for all isolated viruses individually, which calculates the mean score per bird per observation over the 8-day period. Our results revealed that ICPI values ranged between 1.6–2.0 per eight-day observation period for the 20 AOAvV-1 isolates (Table 1) indicating their velogenic nature while ICPI was zero for the two APMV-2 (Table 1) isolates indicating the lentogenic nature of these isolates.

### 3.2. Phylogenetic Analyses

**Figure 2:**
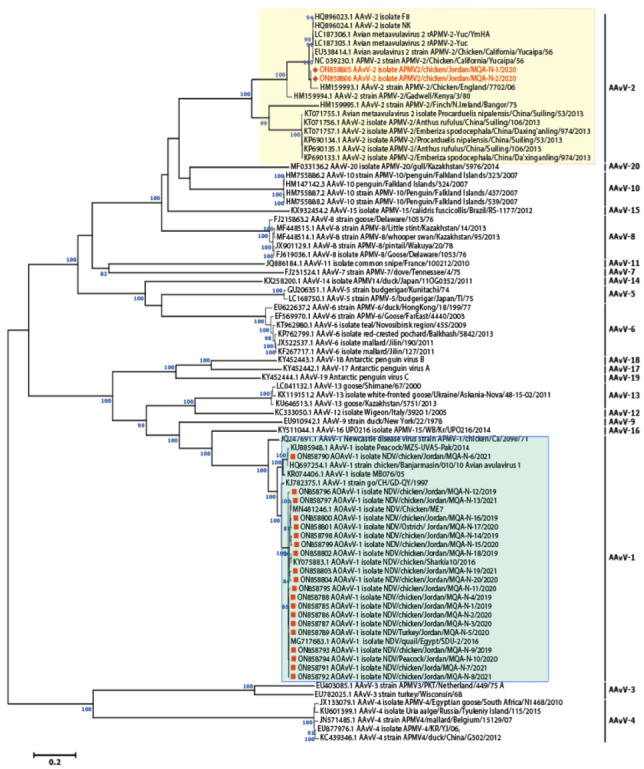
Phylogenomic revealed the clustering of 20 isolates within the AOAVv-1 while two isolates were allocated within AAvV-2/APMV-2. Unrooted phylogenetic trees were generated using the distance-based using maximum likelihood method and MEGA 6 software. Statistical support for tree branches was assessed by bootstrap analysis using 1000 replications of bootstrap re-sampling; numbers above branches indicate neighbor-joining bootstrap values that were ≥80%; the tree is drawn to scale, with branch lengths measured in the number of substitutions per site. The reported AAvV-1 isolates in this study are marked with red square within light green box; however, AAvV-2 isolates are marked with red hexagon labelled within light yellow box.

**Figure 3:**
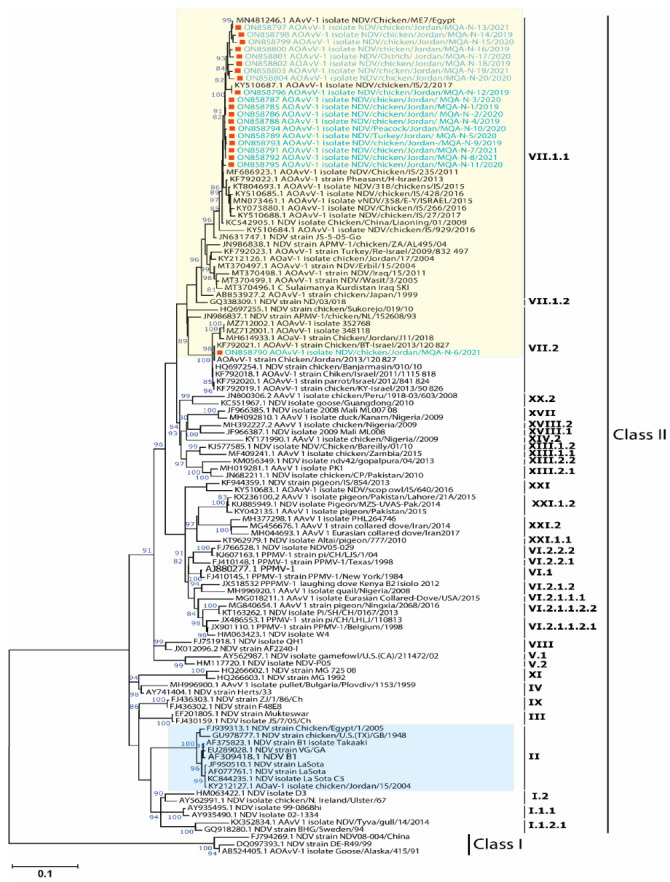
Phylogenetic analysis of the studied AOAvV-1 isolates and their clustering patterns with representative AOAvV-1 isolates. Full-length F-gene (1662 nt)-based phylogenetic analysis of our AOAvV-1 isolates with representative strains of each genotype. Reported isolates clustered in the genotype VII.1.1 of class II. Unrooted phylogenetic trees were generated using the distance-based using maximum likelihood method and MEGA 6 software. Statistical support for tree branches was assessed by bootstrap analysis using 1000 replications of bootstrap re-sampling; numbers above branches indicate neighbor-joining bootstrap values that were ≥80%; the tree is drawn to scale, with branch lengths measured in the number of substitutions per site. The reported AOAvV-1 isolates in this study are marked with red square within yellow box, however, NDV genotype II including LaSota (commonly used vaccine) was labelled within light blue box.

To determine the epidemiological clustering of Jordanian NDV isolates in the current study, representative avian avulaviruses genome sequences from the National Center for Biotechnology Information (NCBI) databases were downloaded and used for phylogenetic and comparative genomic analyses. A Bayesian consensus phylogenetic analysis, which was verified using the neighbor-joining method, 20 isolates in this study clustered within avian avulavirus 1 along with previously reported isolates from Jordan, Israel, Iraq, Egypt, and China (Figure 2) while two isolates were clustered with APMV-2 along with previously reported APMV-2 isolates in Israel (Figure 2). The phylogenomic and clustering pattern of AOAvV-1 isolates revealed that 19 isolates were clustered within genotype VII.1.1 while only one isolate was allocated within genotype VII.2 (Figure 3), showed their close association within the previously reported isolates in Jordan and neighboring countries including Israel, Iraq and Egypt from both commercial and backyard flocks. Interestingly, NDV/Peacock/Jordan/MQA-N-10 isolate was isolated from wild bird indicating the close relationship between wild birds and domesticated birds in NDV epidemiology and evolution.

### 3.3. Nucleotide and Amino Acid Homology

**Figure 4:**
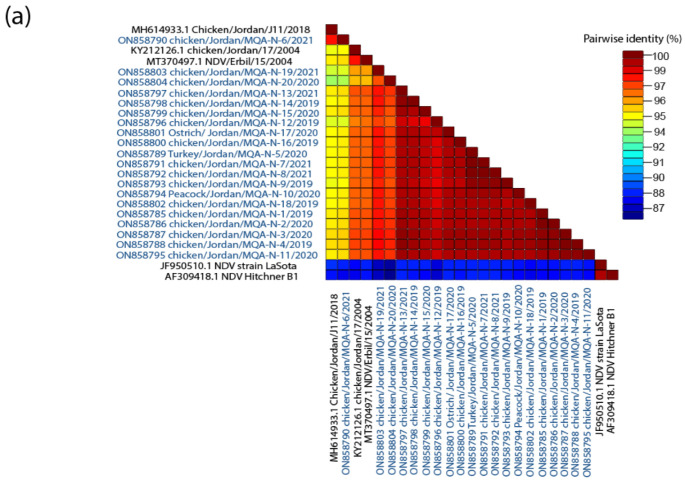

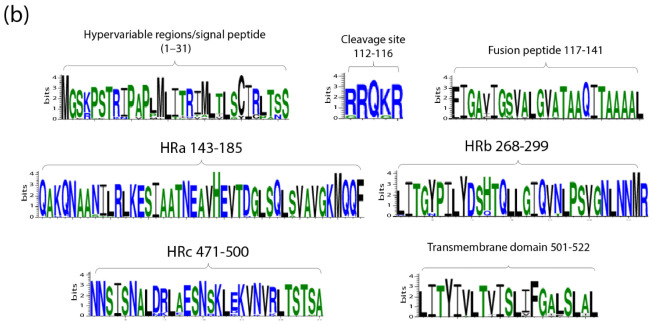
(**a**) The pairwise identities plot of fusion protein sequences aligned by MAFFT and displayed by Sequence Demarcation Tool (SDT) software. (**b**) WebLogo graphs illustrating the amino acid divergence between AOAVv-1 isolates reported in this study compared to LaSota vaccine and previously reported isolates in Jordan.

The level of nucleotide sequence identity between the AOAvV-1 studied isolates ranged between 91% and 99%, and these isolates showed varying degrees of genetic divergence from other representative genotypes of NDV (Figure 4a); however, the identity with the LaSota vaccine strain was 83% (Figure 4a). Meanwhile, all the AOAvV-1 isolates showed 11–13% amino acid difference compared to vaccines that are routinely used in the country (LaSota [genotype II]).

All AOAvV-1 isolates in this study exhibit multiple basic amino acid residues at the cleavage site (F0) of the F protein (Figure 4b), which is a hallmark of velogenic NDV strains [4,45]. The predicted residue analysis of F protein revealed a typical proteolytic cleavage motif ^112^R-R-Q-K-R^116^, characteristic for virulent viruses (Figure 4b). Previous studies have identified six possible glycosylation sites within the F protein that are highly conserved across the majority of AOAvV-1 genotypes. The glycosylation motif Asn-X-Ser/Thr (N-X-S/T, where X is any residue except proline [P] and aspartic acid [D]) was identified in the studied isolates. These sites were identified in the reported AOAvV-1 isolates in this study as follow; ^85^N-RT^87^, ^191^N-N-T^193^, ^366^N-T-S^368^, ^471^N-N-S^473^, and ^541^N-N-T^543^ that are key residues for receptor binding, and crucial amino acids in the hydrophobic core of the stalk [46,47]. Several substitutions were found in the transmembrane region (aa501 to aa521) of the F protein of the AOAvV-1 isolates studied in this study (Figure 4b). In addition, the AOAvV-1 isolates had different alterations in the signal and fusion peptides, and the heptad repeat (HR) regions compared to LaSota and previously reported Jordanian NDV strains (Figure 4b), which might impact on the F protein’s fusogenic activity [45,48,49,50]. 

### 3.4. Deduced Amino Acid Mutations Trend Analyses

In the pathophysiology of the ND, HN glycoprotein starts infection, whereas F glycoprotein facilitates viral attachment and penetration into host cells [2]. Both HN and F proteins stimulate the host immune response and are essential for the production of neutralizing antibodies generated by vaccinations. Antibodies against F proteins have been shown in vivo to be critical in neutralizing ND infectivity [51,52]. Previous studies showed that there are seven major F protein neutralizing epitopes involved in fusion inhibition and neutralization are shown at specific residues 72, 74, 75, 78, 79, 157–171, and 343 for epitopes A1, A2, A3, A4, and A5, respectively. Our results showed that there is an amino acid substitution (H78R) in 17 AOAvV-1 isolates reported in this study. The amino acid residues show that both F1 and F2 are involved in the formation of a single antigenic site vital in the structure and function of the active F epitopes [53].

### 3.5. Antibody Sites Prediction and Immune Pressure

**Figure 5:**
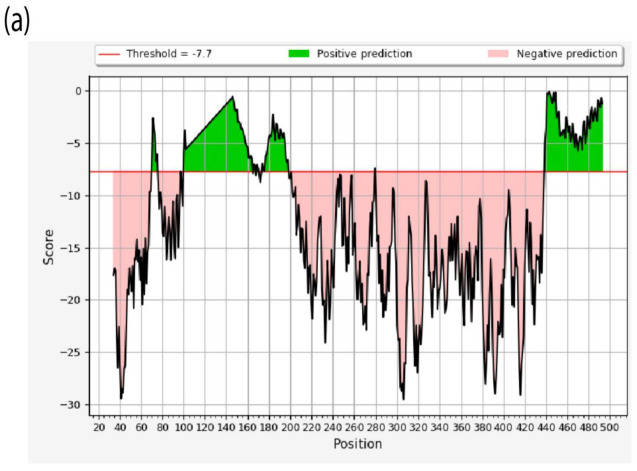

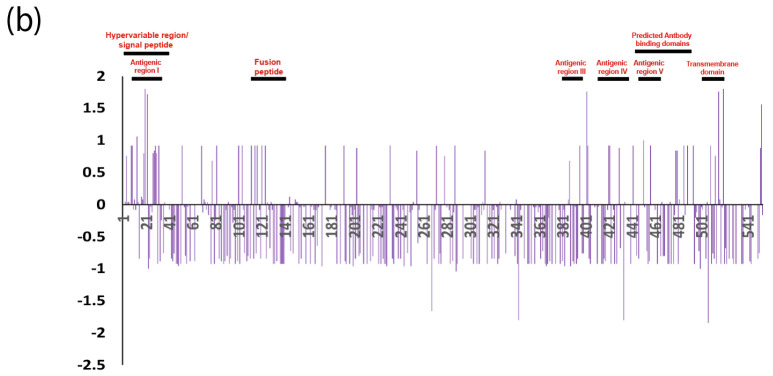
(**a**) Structure-based Antibody Prediction. X-axis contains the position of residues while the y-axis shows the propensity line indicates the threshold value. Regions above the threshold value, shown in green, are representing the residues under positive prediction. (**b**) The cumulative dN/dS of the average synonymous and non-synonymous substitutions moving codon by codon across F protein of AOAVv-1 isolates reported in Jordan including the reported isolates in this study with highlighting the most affected domains (high selective pressure) within the F protein.

We predicted the antibody binding residues and their surface accessibility and antigenicity scores using the BepiPred linear Epitope prediction method, Emini surface accessibility tool, and Kolaskar and Tongaonkar antigenicity, which use epitope scores and immunogenicity predictions through IEDB online (www.iedb.org, accessed on 11 August 2022) facilities. Both BepiPred-2.0 prediction tool and Vaxijen 2.0 tool gave effective antigenic domains; antigenic region I, II, III, IV and V for our AOAvV-1 isolates compared to LaSota vaccine (Table 3). These domains were above antigenicity score (0.8) and surface accessibility score (0.6) suggesting that these amino acid residues could modify the effectiveness of the predicted epitopes, which are speculated for the antigenic differences between these viruses and vaccine. In addition, our analyses showed variable residues that affect the F protein hydrophobic stability (Table 4). Structure-based antibody prediction of the F protein for the AOAvV-1 isolates reported in this study showed different epitope locations (Figure 5a). Mass vaccination has a cumulative effect that plays a role in virus evolution through immune pressure. Our results demonstrated that the cumulative difference between the nonsynonymous substitution rate (dN) and the synonymous substitution rate (dS) for the Jordanian NDV strains were under positive selection at critical sites within the F protein (Figure 5b).

### 3.6. Vaccine Sterility, Safety and Hemagglutination Inhibition Test

The prepared genotype VII.1.1 based inactivated vaccine was sterile and safe as they were free from any bacterial and fungal contaminants. No local or systemic reactions were observed. No clinical signs or mortality were recorded in vaccinated chicks and no pathological lesions were observed by postmortem examination. The post-vaccination antibody titers in the chicks’ sera were determined using HI test with homologous antigens. All chicks had no detectable NDV antibody titers just before vaccination. Similarly, the control group showed no HI antibody titers throughout the study. On the contrary, 3 weeks post vaccination; chickens vaccinated with the genotype VII.1.1 based vaccine showed increasing antibody titer log_2_ 6.73 ± 0.50 compared to LaSota vaccinated chicks showed 4.19 ± 0.95.

### 3.7. Vaccines Efficacy Assessment

**Figure 6:**
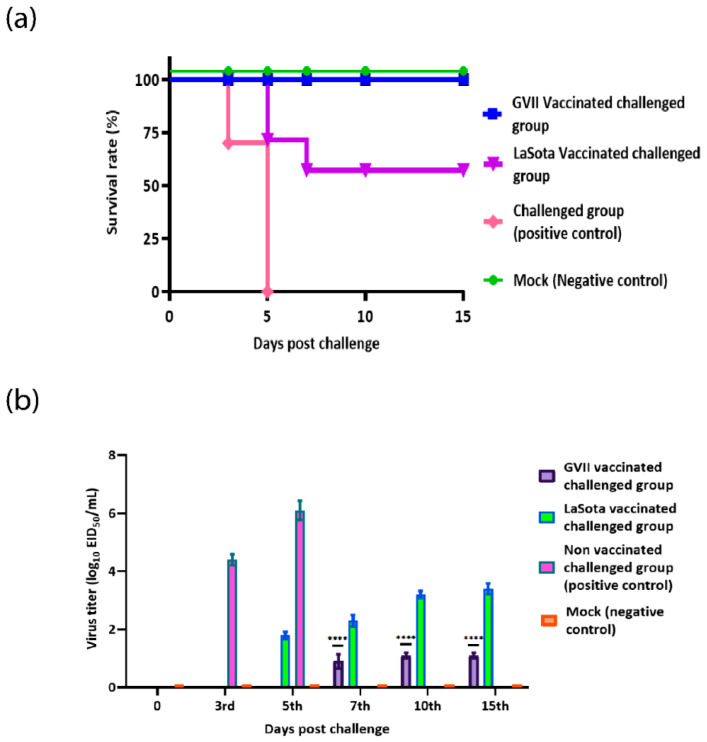
Survival rates and Evaluation of viral shedding. (**a**) Percentage survival rates and (**b**) Viral shedding from oropharyngeal swabs of genotype VII.1.1 and LaSota vaccinated challenged chicks with virulent NDV compared to negative and positive control groups. Bars represent the standard deviation means. **** indicates the level of significance at *p* value < 0.05.

As proved to be highly immunogenic, the protective role of genotype VII.1.1 against virulent viral challenge was compared with LaSota vaccine. The inactivated genotype VII.1.1 vaccine was prepared and used to immunize SPF chicks followed by challenge with homologs virulent NDV strain. Commercial inactivated LaSota vaccine and sterile saline were used as positive and negative immunization controls. Each chick was immunized with a dose of 10^7^EID_50_ via neck subcutaneous injection then challenged with 10^6.5^EID_50_ dose of challenge virus. The clinical symptoms and death of the chicks were recorded every day till 15th days post-challenge. After immunization, all chicks appeared normal before challenge; chicks immunized with either inactivated genotype VII.1.1 or LaSota vaccine did not show any obvious abnormality after challenge; however, non-vaccinated challenged chicks (positive control) showed drowsiness, loss of appetite, apathetic, and row yellow-greenish dilute feces on 2nd day post challenge (dpc) and all chicks died by 5th dpc in this group. These results confirmed that vaccination with genotype VII.1.1 provide complete protection (100%) (Figure 6a) against homologous challenge while LaSota vaccination provided partial protection (60%) (Figure 6a). Clinical signs, representative of ND, started to appear in non-vaccinated challenged group on the 3rd day post-challenge including depression, anorexia, mild respiratory sounds, and oculonasal discharges. Interestingly, all chicks in the non-vaccinated challenged group were died 5 days after infection. On the other hand, clinical signs started to appear in LaSota vaccinated challenged group on the 5th day post challenge and 4 chicks (out of 10) died by the 7th day post challenge (Figure 6a). 

### 3.8. Virus Shedding and Histopathology

The virus shedding data from oropharyngeal swabs were evaluated based on a number of shedders and amount of shedding (EID_50_) at 0, 3rd, 5th, 7th, 10th and 15th days post-challenge. Results of oropharyngeal viral shedding from the vaccinated chicks with genotype VII.1.1 based vaccine showed a significant reduction in the amount of virus shedding compared with LaSota vaccinated group or non-vaccinated challenged group (*p* ≤ 0.05) (Figure 6b), however, there was incomplete prevention for the virus shedding.

**Figure 7:**
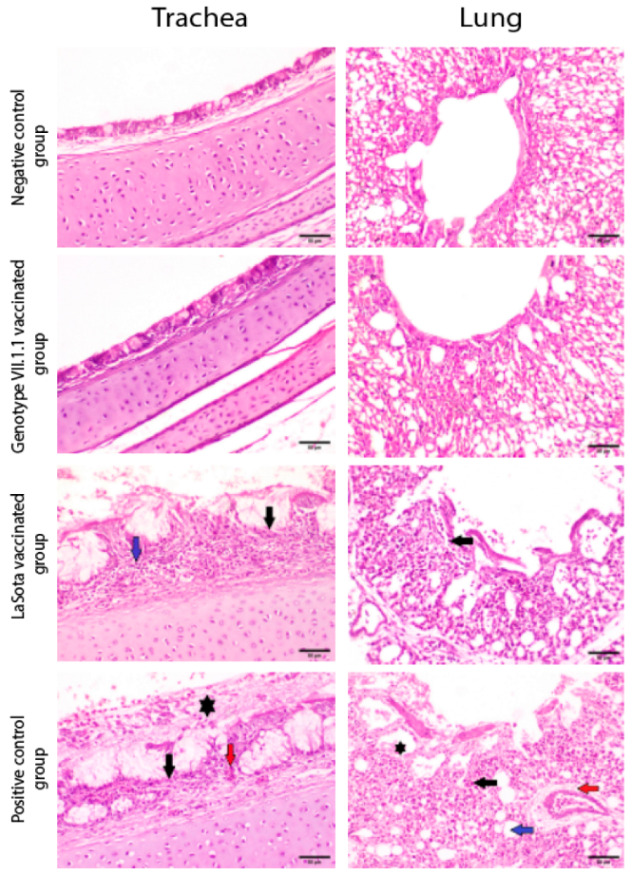
Photomicrographs representing H&E stained sections of tracheas and lungs collected from genotype VII.1.1 and LaSota vaccinated- challenged chicks with virulent NDV compared to mock chicks (negative control) and positive control groups (non-vaccinated challenged chicks). Non-vaccinated- non-challenged chicks and genotype VII.1.1 vaccinated- challenged chicks showing normal histological architecture for Tracheas and normal histological architecture of parabronchus and air capillaries in lungs. On the other hand, LaSota vaccinated challenged chicks showed necrosis of lamina epithelialis and mucosal glands (black arrow) associated with mononuclear cells infiltration in lamina propria (blue arrow) in Trachea and inflammatory cells infiltration (black arrow) in lungs. In addition, non-vaccinated-challenged chicks (positive control group) showing multifocal necrosis of lamina epithelialis (black arrow), congestion (red arrow) and accumulation of mucous exudate in the tracheal lumen (asterisk) in Trachea and showing inflammatory cells infiltration in the air capillaries (black arrow), perivascular edema (red arrow), dilatation of atria (asterisk) and dilatation of air capillaries (blue arrow) in lungs (scale bar 50 µm).

Trachea and lung organs were collected from vaccinated groups either with inactivated genotype VII.1.1 or LaSota based vaccine and non-vaccinated challenged chicks (positive control group) followed by histopathological examination compared with and non-vaccinated non-challenged chicks (negative control group) to assess the level of protection offered by vaccination in face of challenge with a virulent NDV along with the induced histopathological changes. Microscopically, trachea of control non-vaccinated non-challenged chicks and genotype VII.1.1 vaccinated chicks exhibited normal histological structure (Figure 7). On contrary, remarkable histopathological alterations were investigated in tracheal tissues of non-vaccinated challenged chicks (positive control) described by necrosis of lamina epithelialis, mucous secreting glands and mononuclear cells infiltration in lamina propria (Figure 7). Otherwise, moderate changes were noticed in tracheal tissues of LaSota vaccinated chicks; edema in the lamina propria/submucosal layer. The histopathological alterations in the trachea and lungs of different groups are summarized according to their severity in Table S1.

## 4. Discussion

In Jordan and elsewhere, the economic impact of ND on both backyard and commercial poultry is enormous. The recurrence of disease each year, vaccination failures, and potentially circulating of virulent strains in apparently healthy birds constitute a concurrent problem. Concerns about virus evolution, vaccination type utilized to protect birds, and post-vaccine assessment have been proposed several times in previous studies. The anticipated B-cell epitopes and functional domains of F protein in AOAvV-1 isolated from Jordanian birds were compared to those in vaccines to see whether there were any differences between the two groups that might explain the ND vaccination failures. Until now, the AOAvV-1 vaccine has been evaluated only on the basis of empirical cross protection in birds, whereas many of these studies show that ND vaccines with antigenically matched antigens give superior immunity [54,55]. However, such investigations are expensive and time-consuming, and they need extensive field research. Inadequate cold-chain maintenance, insufficient immunization titer, hygienic state, and other variables that might contribute to vaccination failures have all been a source of worry. In this study, we looked at the neutralizing epitopes of F-glycoproteins of enzootic wild ND viruses and vaccine (genotype II) to see if there were any virus variants or recombinants at this antigenic and surface glycoprotein in our AOAvV-1 isolates, which could lead to ineffective vaccination.

In this study, the investigated flocks had varying mortality rate (30–50%) due to putative vNDV infection symptoms such as tracheitis and proventriculus hemorrhage, which are characteristic for velogenic NDV infection [50]. Intracerebral pathogenicity index (ICPI) was used for biological characterization of our isolates reported in this study, which showed that 20 isolates (AOAvV-1) have ICPI ranged between 1.6–2.0 per eight-day observation period. However, only two isolates (APMV-2) showed zero ICPI, suggesting no morbidity or mortality. All samples were negative for other respiratory viruses including influenza viruses and infectious bronchitis virus (IBV). Molecular pathotyping was performed for the AOAvV-1 isolates using the amino acid sequences of the F0 protein cleavage site motifs (residues 112 to 117) because it is a faster and more reliable method than the mean death time (MDT), intravenous pathogenicity index (IVP), and intracerebral pathogenicity index (ICP) tests [56,57]. The majority of virulent NDV strains feature a polybasic cleavage site, which is the primary recognition site for furin (R-X-K/R-R); an intracellular protease presents in most cells that offers an efficient cleavage in a wide variety of tissues, allowing virulent strains to disseminate systemically. While avirulent NDV strains frequently have basic residues at the -1 and -4 positions relative to the cleavage site, which are cleaved by secretory protease. Because avirulent strains cannot be cleaved by furin, their replication is limited to the respiratory and intestinal routes, where secretory protease is available for cleavage.

Our findings revealed that all of the AOAvV-1 isolates had the cleavage site motif ^12^RRQKRF^117^, which is common in velogenic NDV strains. Furthermore, the presence of the phenylalanine (F) residue at position 117, which was detected in our 20 AOAvV-1 isolates, has been characterized as a probable contribution to the neurological consequences [58]. On the other hand, one or two basic residues are detected in the putative F protein cleavage site of APMV-2 isolates (DKPASR↓F), which is similar but not identical to the pattern seen in avirulent NDV strains. Previous research found that APMV-2 replicated in vitro in a wide range of cells without the addition of exogenous protease, and introducing protease did not improve the replication efficiency.

Phylogenetic study of NDV pathotypes is based on the FPCS as well as the hypervariable areas of the F protein [59]. Other ways of classifying NDV strains include genotyping and lineage analysis [60,61]. To date, two classes, I and II, have been identified and each further is classified into three sub-genotypes (1.1.1, 1.1.2, and 1.2) and 21 clades or sub-genotypes (I-XXI), respectively [38,59,60]. Notably, the class II genotype VII viruses are the most commonly reported in ND outbreaks in poultry, pet, and wild birds throughout the world, while class I, which is commonly isolated from waterfowl, shore birds, and some poultry, is less virulent and is exploited for potential vaccine candidates [60,61,62,63,64]. The two membrane-anchored glycoproteins F and HN are possible targets for the immune system response to NDV infection and are also important for cell-binding and infection [65]. ND vaccination failure has been linked to genomic and antigenic variations between field isolates and vaccine strains. These discrepancies result from many accumulated changes in the field strains’ F and HN genes as a result of vaccination pressure [6,63,66]. 

Phylogenetic analysis based on the F gene showed that all 20 AOAvV-1 isolates were related to velogenic strains of NDV; 19 isolates was classified as genotype VII.1.1 (class II) while only one isolate was clustered within genotype VII.2 with close relationship to previously reported isolates in Jordan, Iraq, Israel and Egypt. Moreover, the recently isolated strains are genetically distant from vaccine strains indicating the potential evolution of virulent NDV in the Jordanian poultry sector. Vaccination has been linked to viral evolution in a variety of disease affecting avian, animals and humans. Wild bird strains can spread in a new and more difficult habitat when immunization is not sterilizing. Selective pressure analysis revealed that the circulating Jordanian NDVs are under strong pressure, indicating that vaccination has a role in viral evolution as well as virus adaption in wild birds.

In the present study, the humoral immune response was assessed by HI assay for vaccinated chicks and revealed higher antibody titer; log_2_ 6.73 ± 0.50 and 4.19 ± 0.95 for genotype VII.1.1 and LaSota vaccinated chicks, respectively 3 weeks post vaccination. In addition, our results revealed that the genotype VII.1.1 inactivated vaccine was able to protect the vaccinated chicks from the challenge virus morbidity or mortality. However, the group vaccinated with LaSota inactivated vaccine provide 60% protective efficiency (survival rate). These results were in agreement with previous studies that shown an ND inactivated vaccine must be prepared from current local circulating strains/genotypes [45]. Our results were similar to Miller et al. who observed 100% mortality for non-vaccinated chicks and 100% survival for four weeks-old SPF chicks vaccinated subcutaneously with a single dose of inactivated NDV vaccine after three weeks post-challenge with NDV [55].

While all vaccinated chicks were protected from overt clinical signs and mortality, virus shedding was noted in all the groups vaccinated with the inactivated vaccines. This indicates that these vaccines could only protect against the clinical disease but not against virulent virus infection and replication. Nevertheless, the magnitude and duration of virulent virus shedding in those groups was generally lower than those in the positive control group whose magnitude of the virus shedding was high from day 5 post-challenge. Whether genotype VII.1.1 or LaSota based inactivated ND vaccine was used, the level of virulent challenge virus shed from chicks vaccinated with a homologous vaccine (genotype VII.1.1 based) was significantly lower than that vaccinated with a heterologous vaccine (LaSota based). In consistence, our results demonstrated that the level of specific antibody response against genotype VII.1.1 is higher than that of anti-LaSota response in the vaccinated chicks, which confer a stronger protection against challenge to the immunized chicks, and lead to more efficient control of disease and reduced viral shedding. 

## 5. Conclusions

We have identified that there are at least two types of NDV strains circulating in the country. Importantly, the F protein of the AOAvV-1 isolates were found to map numerous changes that alter the antigenic epitopes and antibody binding domains based on the deduced amino acid analyses. These mutations may significantly affect flocks that have received vaccinations as well as flocks with insufficient immunity in terms of viral immunity and disease dynamics. Therefore, it must be determined if each of these alterations, separately or together, has an impact on the virus’ antigenicity and can have major negative effects on vaccination effectiveness. Continuous genetic and phylogenetic characterization for the circulating AOAvV-1 isolates causing outbreaks are important to understand the AOAvV-1 epidemiology, evolution and to develop novel vaccines and control strategies. The results of the present study confirmed that an inactivated oil-adjuvanted vaccine from the local circulating velogenic AOAvV-1 was efficient to protect the vaccinated birds from morbidity and mortality against the challenge virus. 

## Figures and Tables

**Table 1:** Sampling data and prevalence of Avian Avulaviruses in different geographical regions in Jordan during 2019–2021.

Isolate	Host	Location	ICPI	Accession Number
NDV/chicken/Jordan/MQA-N-1/2019	Backyard chicken	Ajloun	1.8	ON858785
NDV/chicken/Jordan/MQA-N-2/2020	Backyard chicken	Jarash	1.6	ON858786
NDV/chicken/Jordan/MQA-N-3/2020	Backyard chicken	Balqa	1.6	ON858787
NDV/chicken/Jordan/MQA-N-4/2019	Backyard Breeder	Amman	1.7	ON858788
NDV/Turkey/Jordan/MQA-N-5/2020	Turkey	Amman	1.8	ON858789
NDV/chicken/Jordan/MQA-N-6/2021	Layer breeder	Jarash	1.8	ON858790
NDV/chicken/Jordan/MQA-N-7/2021	Broiler breeder	Zaraqa	1.7	ON858791
NDV/chicken/Jordan/MQA-N-8/2021	Layer breeder	Zarqa	1.8	ON858792
NDV/chicken/Jordan/MQA-N-9/2019	Layer breeder	Zarqa	1.7	ON858793
NDV/Peacock/Jordan/MQA-N-10/2020	Peacock	Amman	1.9	ON858794
NDV/chicken/Jordan/MQA-N-11/2020	Layer breeder	Zarqa	1.6	ON858795
NDV/chicken/Jordan/MQA-N-12/2019	Backyard chicken	Madaba	1.8	ON858796
NDV/chicken/Jordan/MQA-N-13/2021	Backyard chicken	Madaba	1.7	ON858797
NDV/chicken/Jordan/MQA-N-14/2019	Backyard chicken	Amman	1.7	ON858798
NDV/chicken/Jordan/MQA-N-15/2020	Layer breeder	Amman	1.9	ON858799
NDV/chicken/Jordan/MQA-N-16/2019	Commercial broiler	Jarash	1.8	ON858800
NDV/Ostrich/Jordan/MQA-N-17/2020	Ostrich	Amman	2.0	ON858801
NDV/chicken/Jordan/MQA-N-18/2019	Commercial broiler	Ajloun	1.8	ON858802
NDV/chicken/Jordan/MQA-N-19/2021	Commercial broiler	Jarash	1.7	ON858803
NDV/chicken/Jordan/MQA-N-20/2020	Commercial broiler	Balqa	1.9	ON858804
APMV2/chicken/Jordan/MQA-N-1/2020	Commercial broiler	Jarash	0	ON858805
APMV2/chicken/Jordan/MQA-N-2/2020	Commercial broiler	Amman	0	ON858806

ICPI: Intracerebral Pathogenicity Index.

**Table 2:** Vaccination regime used to vaccinate the Jordanian poultry flocks.

Age	Vaccination Route	Used Vaccine
0 ^a,b^	In ovo	*VAXXITEK*^®^ (*HVT* + *IBD)*
1 ^a,b^	Coarse spray	Live NDV (Avinew^®^) and live attenuated IBV (Poulvac IB Primer^®^)
14 ^a,b^	Coarse Spray	Live attenuated IBV (IBird^®^) + Live attenuated NDV (Clone 30)
21 ^a,b^	IM	Inactivated NDV + H9N2 + H5N1
28 ^a,b^	Fine Spray	Live attenuated NDV LaSota
49 ^b^	Fine Spray	Live attenuated NDV LaSota
65 ^b^	Eye drop	Live attenuated ILTV
77 ^b^	IM	Inactivated NDV + H9N2
78 ^b^	Fine Spray	Live attenuated NDV LaSota
91 ^b^	SC + IM + Fine spray	Inactivated TRT + IBV + live attenuated IBV (Poulvac IB Primer^®^)
105 ^b^	Fine Spray	Live attenuated ND LaSota
126 ^b^	IM	Inactivated NDV + IBV + IBDV + REO
143 ^b^	IM	Inactivated H9N2 + H5N1
175 ^b^	Fine Spray	Live attenuated NDV (LaSota)

^a^ For broiler flocks; ^b^ For breeder flocks.

**Table 3:** Analysis of mutations in the predicted F protein antigenic domains structure between LaSota vaccine and our AOAvV-1 isolates reported in this study.

Domain	Antigenic Region I (7–30)												Antigenic Region II (196–241)			Antigenic Region III (380–394)			Antigenic Region IV (413–437)			Antigenic Region V (447–460)	
A.A positions	8	9	13	16	17	19	20	22	27	28	29	30	203	231	232	385	386	387	421	422	430	451	457
LaSota (JF950510.1)	K	N	M	T	I	V	A	V	C	P	A	N	A	N	K	T	I	K	K	Q	G	Q	I
AOAvV-1 isolates	R	I	L	I	T	I	M	I	R	L	T	S	T	T	Q	A	L	R	R	H	D	L	V

**Table 4:** Analysis of mutations in the predicted positions contribute to hydrophobic stability of the F protein in our AOAvV-1 isolates reported in this study compared to the LaSota vaccine.

Domain	Variable Residues of Hydrophobic Stability													
A.A positions	69	82	115	124	145	146	192	403	421	430	453	457	486	489
LaSota (JF950510.1)	L	D	G	G	K	Q	K	N	K	G	S	I	R	D
AOAvV-1 isolates	M	E	K	S	K	Q	N	D	R	D	S	V	S	E/K

## Data Availability

Not applicable.

## Supplementary Materials

The following are available online at https://www.mdpi.com/article/10.3390/vaccines10111862/s1, Table S1: Histopathological lesion scores for tracheas and lungs of different experimental groups.
