# Supplementary material for: Evolutionary Trajectories of Avian Avulaviruses and Vaccines Compatibilities in Poultry
Source: Vaccines (Basel). 2022 Nov 3;10(11):1862. doi: 10.3390/vaccines10111862 (PMC9698863; doi:10.3390/vaccines10111862)
Supplement: Supplementary file 1 [file vaccines-10-01862-s001.zip › vaccines-1962907-supplementary.pdf]

Table S1. Histopathological lesion scores for tracheas and lungs of different experimental groups.

	**Histopathological Lesions**	**Negative Control Group**	**Genotype VI.1.1 Vaccinated Group**	**LaSota Vaccinated Group**	**Positive Control Group**
Trachea	Necrosis of lamina epithelialis	0.0 ± 0.0	0.0 ± 0.0	1.6 ± 0.2	2.6 ± 0.5
	Necrosis of mucous secreting glands	0.0 ± 0.0	0.0 ± 0.0	1.4 ± 0.4	2.8 ± 0.6
	Edema in lamina propria/submucosa	0.0 ± 0.0	0.0 ± 0.0	1.6 ± 0.5	2.2 ± 0.2
	Inflammatory cells infiltration	0.0 ± 0.0	0.0 ± 0.0	1.7 ± 0.2	2.6 ± 0.4
Lungs	Inflammatory cells infiltration	0.0 ± 0.0	0.0 ± 0.0	1.8 ± 0.2	2.8 ± 0.2
	Interlobular edema	0.0 ± 0.0	0.0 ± 0.0	1.8 ± 0.6	2.8 ± 0.3
	Dilatation of atria and air capillaries	0.0 ± 0.0	0.0 ± 0.0	1.6 ± 0.3	2.4 ± 0.4
	Exudate in the lumen of parabronchus	0.0 ± 0.0	0.0 ± 0.0	0.6 ± 0.7	1.4 ± 0.2

Data shown as mean ± SD; One-way analysis of variance (ANOVA) were used to determine differences between groups. Statistical significance is shown with values of $p < 0.05$
